# Comparison of Acarbose and Metformin on Albumin Excretion in Patients With Newly Diagnosed Type 2 Diabetes: A Randomized Controlled Trial

**DOI:** 10.1097/MD.0000000000003247

**Published:** 2016-04-08

**Authors:** Qingrong Pan, Yuan Xu, Ning Yang, Xia Gao, Jia Liu, Wenying Yang, Guang Wang

**Affiliations:** From the Department of Endocrinology (QP, YX, NY, XG, JL, GW), Beijing Chaoyang Hospital Affiliated to Capital Medical University; and the Department of Endocrinology (WY), China-Japan Friendship Hospital, Beijing, China.

## Abstract

Increased urinary albumin excretion in diabetes not only signals nephropathy but also serves as a risk marker for cardiovascular disease. The data of MARCH (Metformin and AcaRbose in Chinese as the initial Hypoglycaemic treatment) trial demonstrated that acarbose and metformin were similarly efficacious at lowering blood glucose and blood pressure, as well as improving insulin sensitivity in Chinese patients newly diagnosed with type 2 diabetes mellitus. The purpose of this study was to identify the effects of acarbose and metformin therapy on albumin excretion in MARCH study.

Baseline urine albumin/creatinine ratio (ACR) of 762 newly diagnosed, drug-naïve patients with type 2 diabetes mellitus was measured. Included patients were randomized to receive either acarbose or metformin and followed for 48 weeks. In addition to change in ACR, the estimated glomerular filtration rates (eGFR) and frequency of metabolic syndrome (MetS) were also assessed.

Elevated ACR levels (≥30 mg/g) were present at baseline in 21.9% of all participants. A significant decline in urine ACR was observed in both the acarbose and metformin groups at week 24 and 48 (all *P* < 0.001). The proportion of patients with elevated ACRs was also reduced in both treatment groups at week 24 and 48 compared with baseline values (all *P* < 0.05). The change in urine ACR at week 48 was significantly greater in patients prescribed acarbose than in those prescribed metformin (*P* = 0.01). Both acarbose and metformin significantly decreased the frequency of MetS at week 24 and 48 (both *P* < 0.05). Neither treatment affected eGFR.

In sum, both acarbose and metformin decreased urine ACR levels and reduced the frequency of elevated ACR and MetS in Chinese patients with newly diagnosed type 2 diabetes mellitus without affecting eGFR. After 48 weeks’ intervention, acarbose therapy resulted in a greater reduction in urine ACR compared with metformin.

## INTRODUCTION

Available evidence indicates that increased urinary albumin excretion is associated with an increased risk of both nephropathy and cardiovascular disease in patients with type 2 diabetes mellitus^1–3^ Previous studies suggest that increased urinary albumin excretion correlates with rising glucose levels,^4^ insulin resistance,^5^ elevated blood pressure (BP),^6^ and the presence of metabolic syndrome (MetS).^7^ Both intensive blood glucose control and BP control are effective in reducing urinary albumin excretion.^6,8^ Antihypertensive drugs differ significantly in their albuminuria-lowering capacity despite similar BP-lowering potency, with inhibitors of the renin-angiotensin system being more effective than other antihypertensive agents.^9,10^ However, it is not clear whether the available hypoglycemic drugs have specific anti-albuminuria effects independent of their blood glucose-lowering effects.

Both metformin and acarbose are classical hypoglycemic drugs. Metformin is a first-line agent for the management of type 2 diabetes recommended by international guidelines.^11,12^ In countries such as China, however, where rice forms a major component of the diet and the dietary contribution of carbohydrates is high, acarbose, which reduces the rate of carbohydrates absorption, is prescribed popularly. The MARCH study (Metformin and Acarbose in Chinese as the initial Hypoglycemic treatment), a randomized, open-label, multicenter clinical trial, compared the efficacy and safety of acarbose versus metformin in drug-naïve patients with newly diagnosed type 2 diabetes mellitus in China.^13,14^ We previously reported that acarbose and metformin were similarly efficacious at lowering glycated hemoglobin A1c (HbA1c) and BP, as well as improving insulin sensitivity after 24 and 48 weeks of therapy in the MARCH study.^13,14^

The purpose of this study was to analyze the effects of acarbose and metformin on urinary albumin excretion as measured by the albumin/creatinine ratio (ACR) in the MARCH study. The frequency of MetS was also assessed. It has been reported that the reduction of urinary albumin excretion by hypoglycemic strategies is associated with the amelioration of glomerular hyperfiltration in early type 2 diabetes mellitus.^15,16^ Therefore, we also assessed the estimated glomerular filtration rates (eGFR).

## MATERIALS AND METHODS

### Research Design and Study Participants

This multicenter, randomized controlled study was registered with the Chinese Clinical Trial Registry, number ChiCTR-TRC-08000231 (www.chictr.org.cn/index.aspx) and was conducted at 11 hospital centers in China. All subjects provided written informed consent. The study received approval from the Ethics Committee from each clinical site (China-Japan Friendship Hospital, Beijing, China; Shanxi Province People's Hospital, Taiyuan, China; The First Hospital of China Medical University, Shenyang, China; West China Hospital, Sichuan University, Chengdu, China; Xiangya Second Hospital of Central South University, Changsha, China; Xijing Hospital, Fourth Military Medical University, Xi’an, China; The Third Affiliated Hospital of Sun Yatsen University, Guangzhou, China; Shanghai jiaotong University Affiliated Sixth People's Hospital, Shanghai, China; Chinese People's Liberation Army General Hospital, Beijing, China; Gansu Provincial Hospital, Lanzhou, China; Beijing Chaoyang Hospital Affiliated to Capital Medical University, Beijing, China). All participants were diagnosed with type 2 diabetes mellitus within the 12 months before study participation. Diagnosis was made based on World Health Organization diabetes criteria of 1999. Full details of the inclusion criteria, exclusion criteria, randomization and masking have been published.^13,14^ Pertinent to the current analysis, the following exclusion criteria should be noted: a history of renal disease with a plasma creatinine concentration of ≥133 μmol/L (1.5 mg/dL); cardiac diseases (ie, a history of unstable angina or myocardial infarction within the previous 6 months or New York Heart Association class III or IV congestive heart failure); uncontrolled hypertension (systolic pressure ≥160 mmHg or diastolic pressure ≥95 mmHg); and urinary infection (urine leukocytes >5/high-power field in a standard urine analysis).

All eligible subjects began a 4-week run-in period during which they were given lifestyle instructions and diet counseling. Patients were then randomly assigned to receive either 1500 mg/day metformin hydrochloride (Beijing Double Crane Pharma, Beijing, China) or 300 mg/day acarbose (Bayer Healthcare, Beijing, China). The study period was 48 weeks.

The primary endpoints were reduction in ACR after 48 weeks’ intervention. Secondary endpoints included change in eGFR, the proportion of patients with ACR ≥30 mg/g or with MetS, all measured at baseline, 24 weeks, and 48 weeks.

### Outcome Measures

Serum and urine creatinine concentrations were measured enzymatically, and urine albumin concentration was measured via an immunoturbidimetric assay. Albuminuria was assessed using a spot urine test of albumin and creatinine in a morning sample. Urine albumin and creatinine were measured at baseline, week 24, and week 48. The ACR was used to define 3 categories of albuminuria: normal (<30 mg/g), microalbuminuria (30–300 mg/g), and macroalbuminuria (≥300 mg/g).^17^ In this report, the term “elevated ACR” included both the micro- and macroalbuminuria groups.

As an indicator of kidney function, eGFR was calculated using a formula developed from an adaptation of the Modification of Diet in Renal Disease (MDRD) equation base on data from Chinese chronic kidney disease patients^18^: eGFR (mL/min/1.73 m^2^) = 175 × (serum creatinine in mg/dL)^−1.234^ × (age)^−0.179^ × 0.79 (if female).

In addition to type 2 diabetes mellitus, 4 other MetS components based on the National Cholesterol Education Program (NCEP) criteria were assessed.^19^ Those components were waist circumference ≥90 cm in men or ≥80 cm in women; BP ≥130/85 mmHg or use of an antihypertensive medication; serum high-density lipoprotein (HDL) cholesterol levels <1.04 mmol/L in men or <1.30 mmol/L in women or specific treatment for HDL cholesterol; and serum triglyceride levels ≥1.7 mmol/L or specific treatment for elevated triglycerides. MetS was diagnosed if the patient had type 2 diabetes together with ≥2 of the above-mentioned MetS components.

The following formulae were used to calculate homeostasis model assessment-insulin resistance (HOMA-IR) and homeostasis model assessment-β cell function (HOMA- B):

HOMA-IR = fasting insulin × fasting plasma glucose/22.5

HOMA-B = 20 × fasting insulin/(fasting plasma glucose–3.5)

### Statistical Analyses

All analyses were performed using SPSS 17.0 statistical software (SPSS Inc., Chicago, IL). Continuous variables were presented as either mean ± standard deviation (SD) or median (interquartile range). Categorical variables were described as numbers (%). The 2-tailed *t* test or Kruskal-Wallis test was used to compare the parameters between the 2 different treatment groups. Categorical variables were compared using the *χ*^2^ test. Changes in parameters from the baseline values within treatment groups were evaluated using the 2-tailed paired *t* test (the continuous variables in normal distribution) or the nonparametric Wilcoxon signed-rank test (the skewed continuous variables). Spearman correlation was used to analyze the correlation of the changes in ACR and the changes in selected parameters after 48 weeks’ treatment. A value of *P* < 0.05 was considered to be statistically significant.

## RESULTS

### Baseline Assessments

From November 8, 2008 to June 27, 2011, we screened 1099 patients and randomly allocated 788 to the 2 treatments. Four withdrew consent before drug intervention. A total of 784 patients commenced study drug (393 metformin and 391 acarbose). The study flowchart has been published elsewhere.^13^

The current report analyzed 762 participants entering the study with valid urine ACR measurements (excluded urinary infection) before randomization. Among all participants, the median ACR was 11.74 mg/g. Overall, 78.1% of the study participants had normal ACR levels (<30 mg/g), 21.9% had elevated ACR levels (≥30 mg/g), and 0.5% had macroalbuminuria (ACR ≥300 mg/g). Multi-liner regression analysis with Log-transformed ACR (ACR was a skewed continuous variable) as the dependent variable was performed. The independent variables included systolic and diastolic BP, HbA1c, low-density lipoprotein cholesterol (LDL-C), triglyceride level, and HOMA-IR. Urine albumin excretion was independently associated with diastolic BP, HbA1c, and HOMA-IR. Systolic BP, LDL-C, and triglyceride were excluded from the final model because they were not significantly related in the presence of the above variables.

There were no significant differences in demographic and clinical characteristics between acarbose and metformin groups (all *P* > 0.05; Table 1). Moreover, baseline urine ACR level, eGFR, and metabolic parameters, including body mass index, waist circumference, BP, HbA1c, fasting plasma glucose (FPG), 2 hour post-challenge plasma glucose (PPG), HOMA-IR, HDL-C, LDL-C and triglyceride level showed no significant differences between the 2 groups (all *P* > 0.05, Table 2). There were also no significant differences in the proportions of patients with MetS and elevated ACR between 2 groups at baseline (both *P* > 0.05, Table 2).

**TABLE 1:**
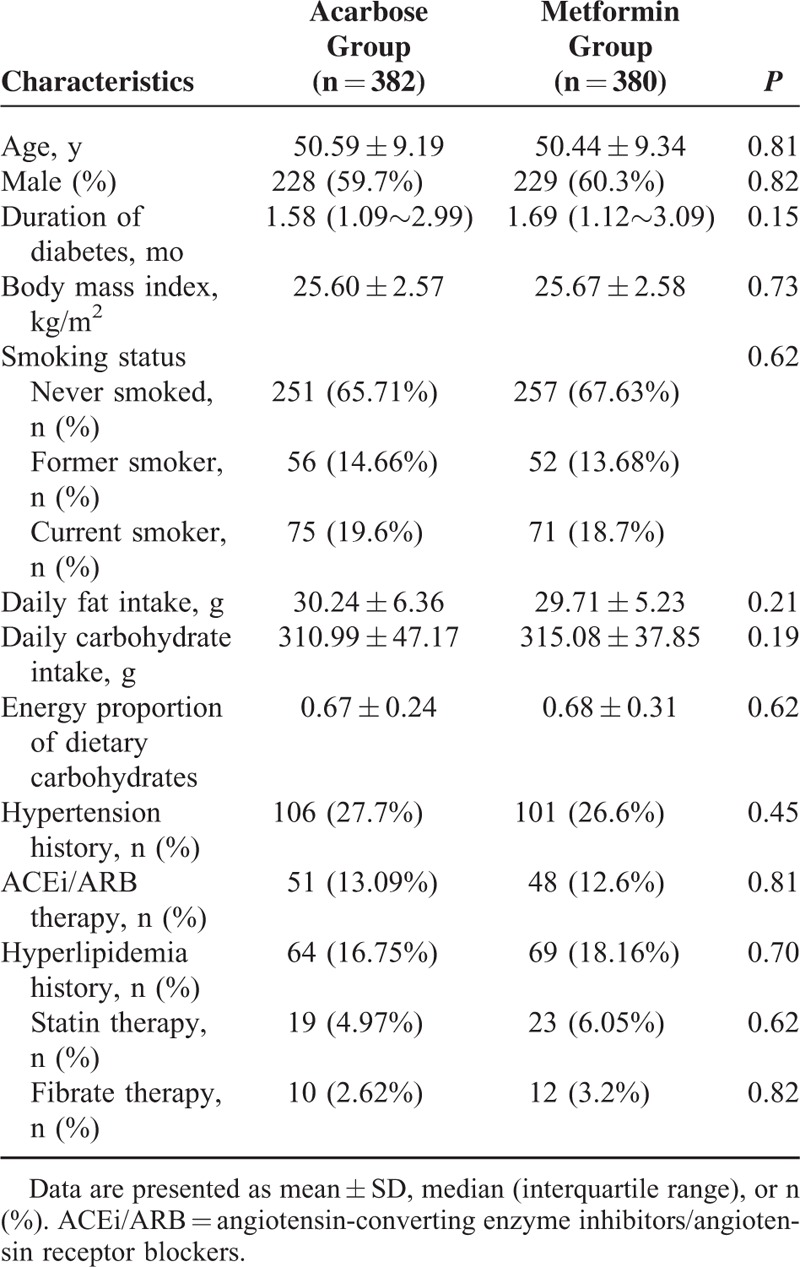
Baseline Characteristics in 2 Treatment Groups

**TABLE 2:**
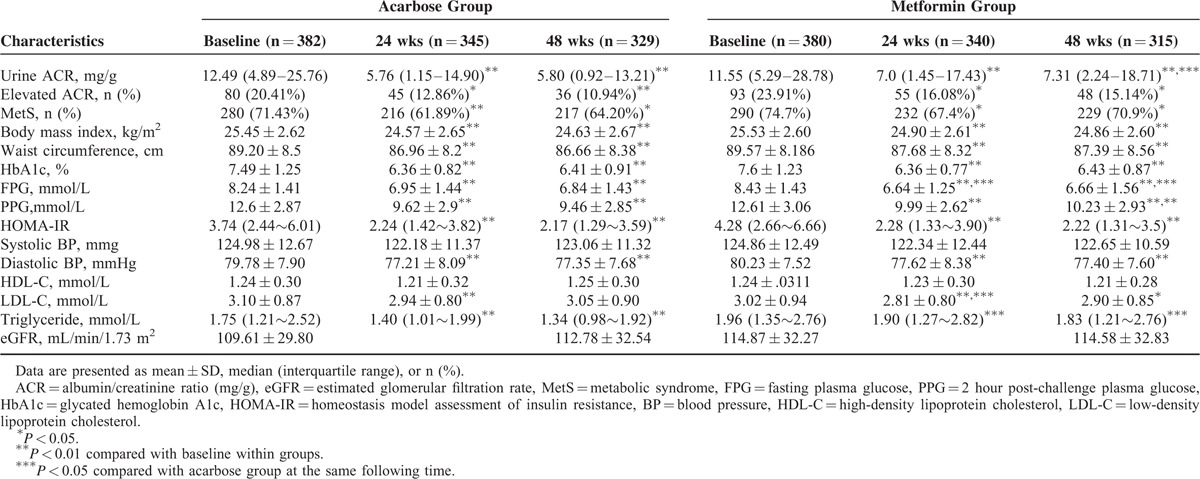
Urine ACR, eGFR, and Metabolic Parameters Before and After Treatment With Acarbose or Metformin in Newly Diagnosed Type 2 Diabetes Mellitus Patients

### Changes in ACR Following Acarbose or Metformin Therapy

Of the study participants with available baseline evaluations, 685 and 644 had valid ACR measurements performed at week 24 and 48, respectively. A significant decline in urine ACR was observed in both the acarbose and metformin groups at week 24 and 48 (all *P* < 0.001). The proportion of patients with elevated ACRs was also reduced in both treatment groups at week 24 and 48 compared with baseline values (all *P* < 0.05; Table 2).

**FIGURE 1:**
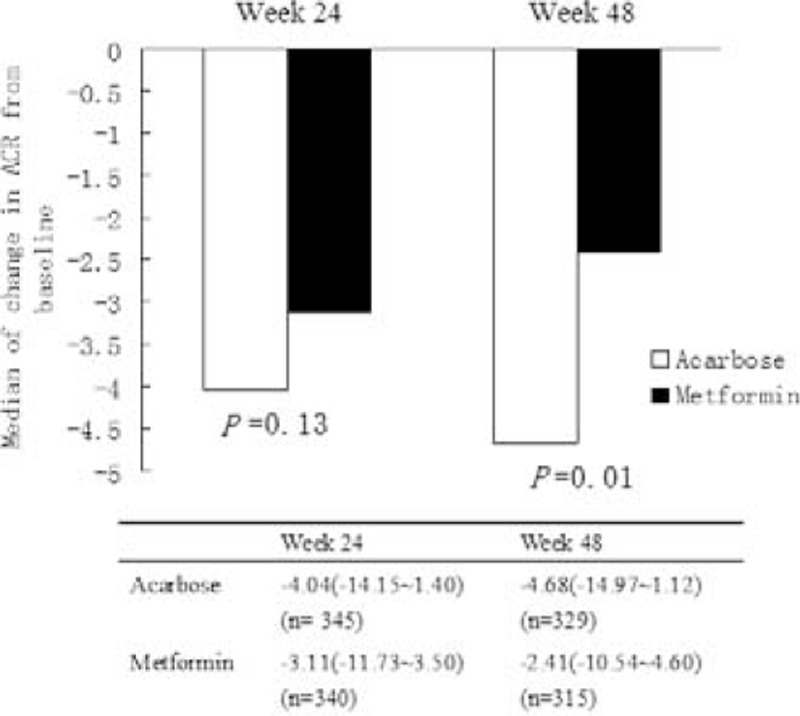
Absolute change in albumin/creatinine ratio (ACR, mg/g) from baseline values in acarbose and metformin treatment groups. Data are presented as median (interquartile range). CR, albumin/creatinine ratio (mg/g).

In comparison between acarbose and metformin groups after intervention, urine ACR level in acarbose treatment group was significantly lower than that in metformin treatment group at week 48 (*P* < 0.05; Table 2). Furthermore, the reduction of urine ACR (absolute change in ACR from baseline value) was significantly greater in patients taking acarbose than in those taking metformin at week 48 (*P* = 0.01; Figure 1).

### Change in MetS Frequency and MetS Components Following Acarbose or Metformin Therapy

The prevalence of MetS in study participants with newly diagnosed type 2 diabetes mellitus was 74.01% at baseline. As showed in Table 2, both acarbose and metformin treatments significantly decreased the frequency of MetS at week 24 and 48 (all *P* < 0.05). Both acarbose and metformin treatments significantly decreased HbA1c, FPG, PPG, HOMA-IR, diastolic BP, LDL-C, body mass index, and waist circumference at week 24 and 48 (all *P* < 0.05). Neither treatment affected HDL-C. The serum triglyceride level was significantly decreased in study patients receiving acarbose treatment at week 24 and 48 (both *P* < 0.001), but not in patients receiving metformin treatment at week 24 and 48 (both *P* > 0.05).

In comparisons between acarbose and etformin groups after 48 weeks’ intervention, triglyceride and PPG levels in patients taking acarbose were lower than those taking metformin, whereas FPG was higher in patients taking acarbose than those taking metformin. There were no differences of the frequency of MetS as well as HbA1c, HOMA-IR, LDL-C, HDL-C, BP, body mass index, and waist circumference between the 2 treatment groups at week 24 and 48 (Table 2).

### Change in eGFR Following Acarbose and Metformin Therapy

Neither treatment affected eGFR at the end of the study (Table 2). There was no difference in eGFR between the acarbose and metformin groups after 48 weeks’ treatment.

### Correlation Between the Change in Urine ACR and the Changes in Selected Study Parameters After 48 Weeks of Treatment

In acarbose group, there were significant correlations between the change in urine ACR and the changes in HbA1c, diastolic BP, and triglyceride after 48 weeks of treatment. In contrast, only the changes in HbA1c and diastolic BP were significantly associated with the change in urine ACR in metformin group (Table 3).

**TABLE 3:**
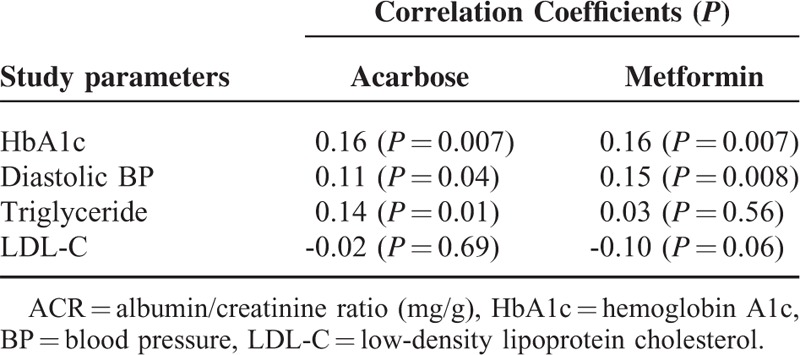
Correlation Between the Change in Urine ACR and the Changes in Selected Study Parameters After 48 Weeks of Treatment

## DISCUSSION

In this study, both acarbose and metformin decreased urine ACR levels as well as the frequency of elevated ACR over a 48-week study period. Both acarbose and metformin showed some beneficial effects on the frequency and components of MetS. Further, ACR levels were correlated with hypertension, hyperglycemia, and insulin resistance in the MARCH study, and that correlation was also shown in other studies of individuals with type 2 diabetes mellitus.^4–7^ It is possible that the effects of both acarbose and metformin on urine ACR are related to decreased blood glucose levels, BP, and degree of insulin resistance.

Metformin has previously been shown to decrease urinary albumin excretion in patients with type 2 diabetes mellitus.^20^ However, in the ADOPT study (A Diabetes Outcomes Prevention Trial), the largest study to address the changes of urine albumin excretion with rosiglitazone, metformin, and glyburide monotherapies, metformin therapy did not change albuminuria in the first year. Furthermore, raised urine ACR slowly increased over the subsequent 4-year period.^21^ Two other studies demonstrated that metformin (either 500 mg/day or ≤850 mg/day) was ineffective in changing albuminuria in a shorter time.^22,23^ The discrepancies regarding motorman's effects on albuminuria could be because of differences in study design (eg, duration of diabetes and hypertension frequency at baseline, metformin dose, ethnicity, etc). It should be noted that: the frequencies of hypertension and either angiotensin-converting enzyme inhibitors (Ace) or angiotensin receptor blockers (ARB) usage at baseline in the ADOPT study were 78.26% and 33.14%, respectively, and both were much lower in the present study; and the duration of diabetes in the ADOPT study was longer than that in the present study. Specifically, more than half the participants in the ADOPT study had been diagnosed with type 2 diabetes for > 1 year. Therefore, it is possible that the increased urinary albumin excretion in participants in the ADOPT study was more advanced and potentially irreversible. If that hypothesis is even partially correct, metformin's reduction in urine albumin excretion may be more effective in individuals in the early stages of albuminuria and those with a lower risk of developing albuminuria.

We observed that acarbose had stronger impact on urine ACR compared with metformin at week 48. The precise mechanism(s) remain(s) unknown. It cannot be explained by differences in BP, glucose, or insulin resistance. The study authors observed a significant decline in plasma triglyceride concentration in the acarbose group but not in the metformin group. In addition to BP and glucose levels, elevated plasma triglyceride concentration has been reported to be associated with a decline in renal function and progression of albuminuria in patients with type 2 diabetes mellitus.^24,25^ Another study shows that fatty acids derived from triglycerides could pass through the glomerulus, resulting in progressive glomerular and tubule damage.^26^ Furthermore, both the Fenofibrate Intervention and Event Lowering in Diabetes study (FIELD) and the Diabetes Atherosclerosis Intervention Study (DAIS) report reductions in triglycerides and urine albumin excretion by fenofibrate.^27,28^ It is possible that the higher efficacy of acarbose on the attenuation of hypertriglyceridemia may be associated with the greater reduction in urine ACR in the acarbose group. It should also be noted that the relative energy proportion of dietary carbohydrates consumed by the participants in MARCH study is higher than that recommended by international guidelines (45%–65%), with mean 67% at baseline, 66% at 24 weeks, and 68% at 48 weeks.^14^ Acarbose, as an alpha-glucosidase inhibitor, which reduces the rate of carbohydrate absorption, probably displays more protective effects in high dietary carbohydrate intake populations, such as Chinese.

The main limitation in this study was, for ethical reasons, the lack of a placebo group. Second, the participants in this study were all Chinese; Chinese, other than people in the western country, have certain genetic backgrounds and favor high carbohydrate diet. Further research in other countries would be necessary to ensure the results apply to other populations. A third limitation was the short follow-up period. Whether the findings in this study could have been translated into microvascular protection and, perhaps more importantly, macrovascular protection remains to be investigated, as MARCH was not designed to assess these long-term outcomes.

In conclusion, the key finding was that both acarbose and metformin decreased urine ACR and reduced the frequency of elevated ACR and MetS in Chinese patients with newly diagnosed type 2 diabetes mellitus without affecting eGFR. In addition, acarbose monotherapy showed a greater reduction in urine ACR compared with metformin after 48 weeks’ intervention.
